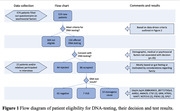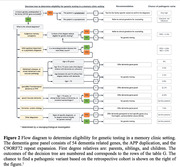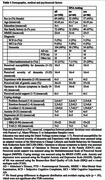# Patients’ perceptions and considerations regarding DNA testing in a memory clinic: DNA‐ABOARD

**DOI:** 10.1002/alz.091187

**Published:** 2025-01-09

**Authors:** Jetske van der Schaar, Sven J van der Lee, Eva Asscher, Wiesje M. van der Flier, Yolande A.L. Pijnenburg, Annelien L. Bredenoord, Mariette A. van den Hoven, Ellen M.A. Smets, Leonie N.C. Visser

**Affiliations:** ^1^ Alzheimer Center Amsterdam, Neurology, Vrije Universiteit Amsterdam, Amsterdam UMC location VUmc, Amsterdam Netherlands; ^2^ Amsterdam Neuroscience, Neurodegeneration, Amsterdam Netherlands; ^3^ Genomics of Neurodegenerative Diseases and Aging, Human Genetics, Vrije Universiteit Amsterdam, Amsterdam UMC, Amsterdam Netherlands; ^4^ Department of Ethics, Law and Humanities, Amsterdam UMC, Amsterdam Netherlands; ^5^ Department of Epidemiology and Data Science, Vrije Universiteit Amsterdam, Amsterdam UMC, Amsterdam Netherlands; ^6^ Alzheimer Center Amsterdam, Neurology, Vrije Universiteit Amsterdam, Amsterdam UMC, Amsterdam Netherlands; ^7^ Amsterdam Neuroscience, Neurodegeneration, Amsterdam, Noord‐Holland Netherlands; ^8^ Erasmus School of Philosophy, Erasmus University Rotterdam, Rotterdam Netherlands; ^9^ Amsterdam Public Health, Quality of Care, Personalized Medicine, Amsterdam Netherlands; ^10^ Medical Psychology, Amsterdam UMC location AMC, University of Amsterdam, Amsterdam Netherlands; ^11^ Amsterdam Public Health, Quality of Care, Amsterdam Netherlands; ^12^ Division of Clinical Geriatrics, Center for Alzheimer Research, Department of Neurobiology, Care Sciences and Society, Karolinska Institutet, Stockholm Sweden; ^13^ Department of Medical Psychology, Amsterdam UMC, University of Amsterdam, Amsterdam Netherlands

## Abstract

**Background:**

Data‐driven criteria for DNA testing were implemented in routine care of Alzheimer Center Amsterdam. We aimed to explore patients’ perspectives and considerations regarding their decision to (not) be tested for a monogenic cause of their disease.

**Methods:**

In this mixed method study, 150 of 519 new patients visiting Alzheimer Center Amsterdam who fulfilled the criteria were offered DNA‐diagnostics: 86(57%) accepted, 64(43%) did not. In 65/86(76%) cases results were negative, in 14/86(16%) a monogenic cause and in 7/86 (8%) variants of uncertain significance were found (*Figure 1)*. Sex, age, education, family history of dementia, MMSE and diagnosis were retrieved. Before discussing DNA testing, patients completed a questionnaire assessing perceived risk of a genetic cause, susceptibility for, severity of, and experience with dementia, social support, coping strategies, anxiety, depression, and quality of life. Differences between those who did and did not consent to DNA testing were calculated using Pearson’s χ², Mann‐Whitney U or Independent Samples t‐tests; p‐values were FDR‐adjusted. A subset of 22 patients and/or relatives participated in semi‐structured interviews on motivations and considerations regarding their decision. Verbatim transcripts were analyzed inductively using MAXQDA‐software.

**Results:**

Participants (n = 150) were 46% female and aged 61±8 (MMSE = 22±6, 5 Subjective Cognitive Decline, 9 MCI, 104 dementia [69 AD, 13 FTD, 22 other], 32 other/undetermined). Adjusted for multiple testing, no demographic, medical or psychosocial factors were associated with the decision on DNA testing (all *p*>0.05). Twenty‐one interviewees who agreed to DNA testing were generally motivated by obtaining information on heredity for their relatives, contributing to scientific research into a treatment, and gaining insight in the cause of their disease. One patient had blood stored, allowing their children to test it when opportune. The majority decided quickly, mostly on intuitions (e.g., responsibility towards relatives) rather than facts (e.g., information on risk and consequences). They expected to feel reassured or relieved by negative test results, and sad or worried if a genetic cause was found.

**Conclusions:**

Over half of memory clinic patients eligible for DNA‐testing wanted to be tested. This decision was unrelated to demographic, medical or psychosocial characteristics, and mostly based on intuition and considerations regarding family.